# Overstrung Nerves

**Published:** 1891-12

**Authors:** 


### OVERSTRUNG NERVES.

It is not the work but the worry which kills. There is no tonic for the body like regular work of the mind, though this is unfortunately not often appreciated or not allowed by the physicians to whom anxious mothers take their growing daughters. There is nothing so sure to steady the nerves of the fretful and excitable child as regular school work in the hands of a real teacher. Many a child who is celebrated for dangerous fits of temper at home becomes entirely transformed under the influence of such a school, till her nearest relatives would not recognize her if they should ever take the time and trouble to visit the school-room. I do not mean a school-room full of competitive examinations, of "marks," and of irrelevant inducements to make the child commit to memory a mass of unrelated and undigested facts. I mean one where, without any inducement but the natural desire for knowledge, which is all-sufficient with any American child if it be rightly directed, you find steady and well ordered labor, without haste, though not without rest, and honest, thorough, and pleasurable work.

We may learn a lesson from this fact—for it is no theory—of the effect of regular work on our tired nerves, and wise shall we be if we apply it. Even the most consistent homœopathic physician could not object to this kind of tonic ; though he would tell you, and truly, that tonics are worse than of no use for overworked nerves.

